# Supplementary material for: Temperature and volumetric effects on structural and dielectric properties of hybrid perovskites
Source: Nat Commun. 2024 Aug 31;15:7571. doi: 10.1038/s41467-024-51396-5 (PMC11365980; doi:10.1038/s41467-024-51396-5)
Supplement: Supplementary file 1 — Supplementary Information [file 41467_2024_51396_MOESM1_ESM.docx]

**Supplementary Information**

**Temperature and volumetric effects on structural and dielectric properties of hybrid perovskites**

Andrzej Nowok, Szymon Sobczak, Kinga Roszak, Anna Z. Szeremeta, Mirosław Mączka, Andrzej Katrusiak, Sebastian Pawlus, Filip Formalik, Antonio José Barros dos Santos, Waldeci Paraguassu, and Adam Sieradzki

**Supplementary Figures**


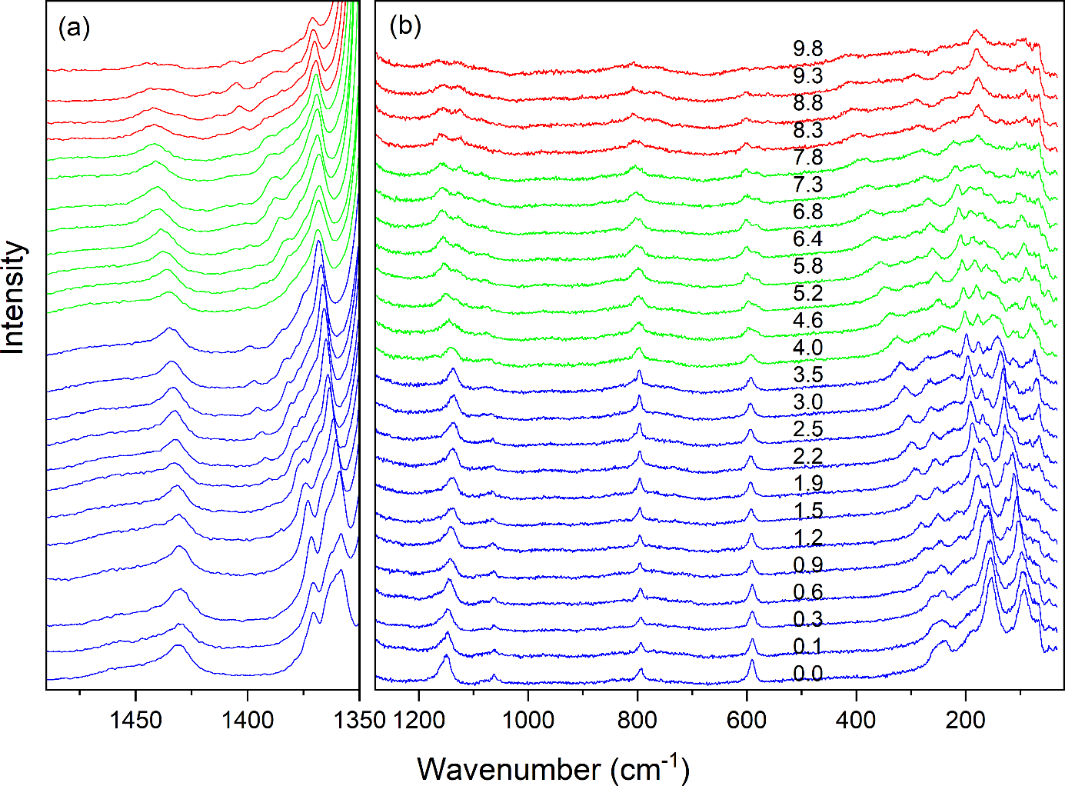


**Supplementary Figure 1.** **High-pressure Raman spectra of FMDMn.** Raman spectra of FMDMn measured during room-temperature compression. Blue, green and red color correspond to phase II, III, and IV, respectively. Source data are provided as a Source Data file.


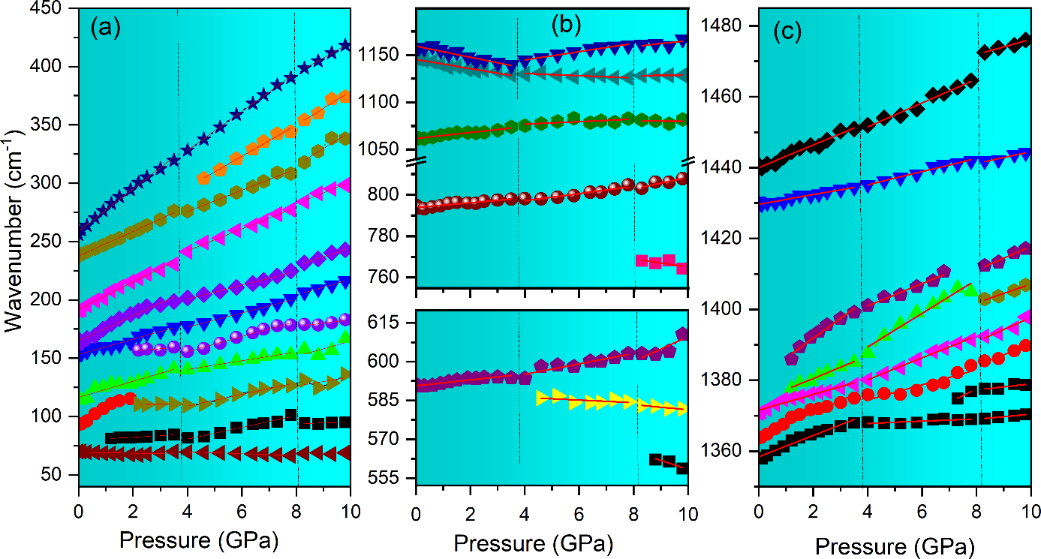


**Supplementary Figure 2. Pressure dependence of the Raman modes in FMDMn.** Pressure dependence of the Raman modes measured during room-temperature compression. Experimental data are presented as color points. Solid lines are linear fits on the data to *ω*(*p*) = *ω*_0_+*αp*. Vertical dashed lines correspond to the phase transition pressures. Source data are provided as a Source Data file.


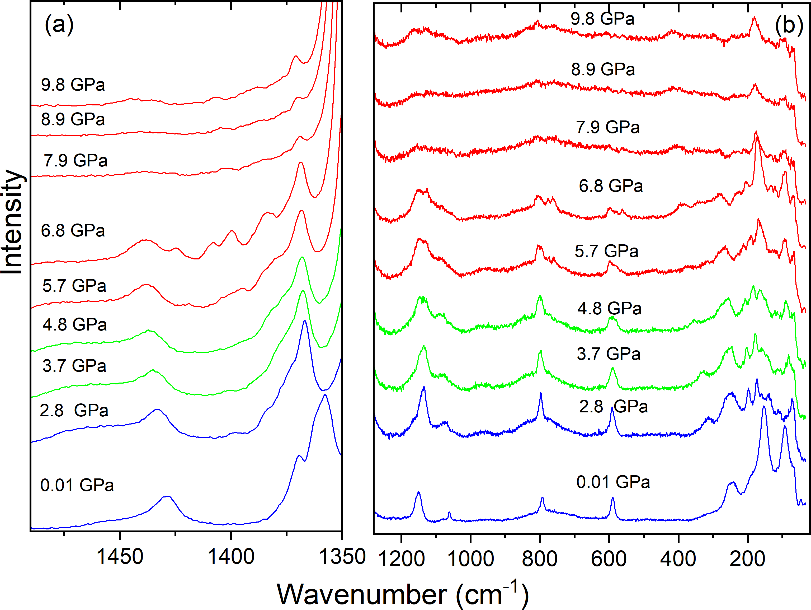


**Supplementary Figure 3. Raman measurements on decompression.** Raman spectra of FMDMn collected under various pressures during room-temperature decompression cycle. Blue, green and red color correspond to phase II, III, and IV, respectively.


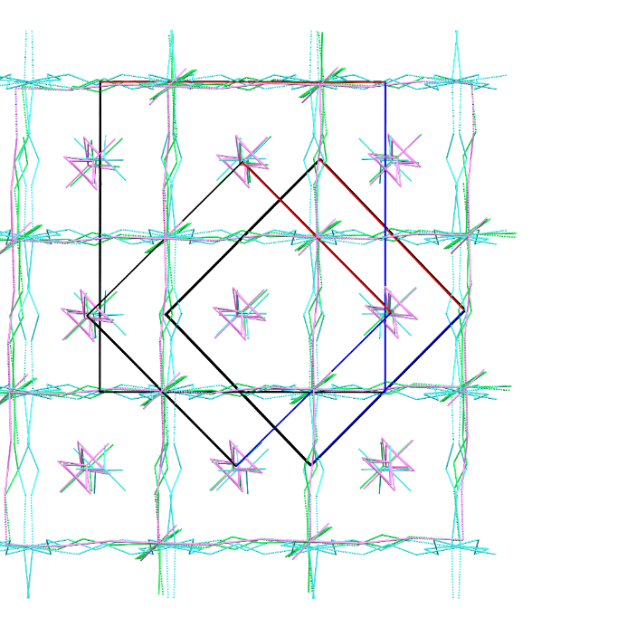


**Supplementary Figure 4.** **Structural analysis for AceMn.** The unit cell of phases I and III of AceMn superimposed on the unit cell of ambient-pressure phase I.


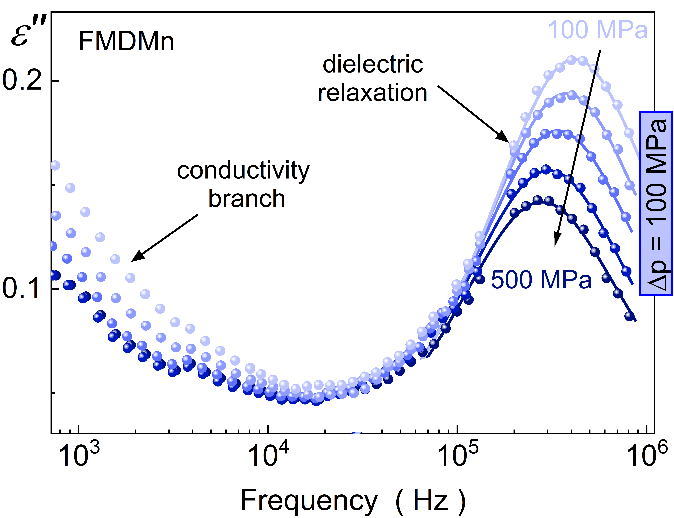


**Supplementary Figure 5.** **High-pressure dielectric data for FMDMn.** Exemplary room-temperature *ε"*(*f*) spectra collected between 100 and 500 MPa for FMDMn fitted with the Havriliak-Negami function in the vicinity of relaxation peak. Experimetal data and fitting curves are presented as color points and lines, respectively. Source data are provided as a Source Data file.

**
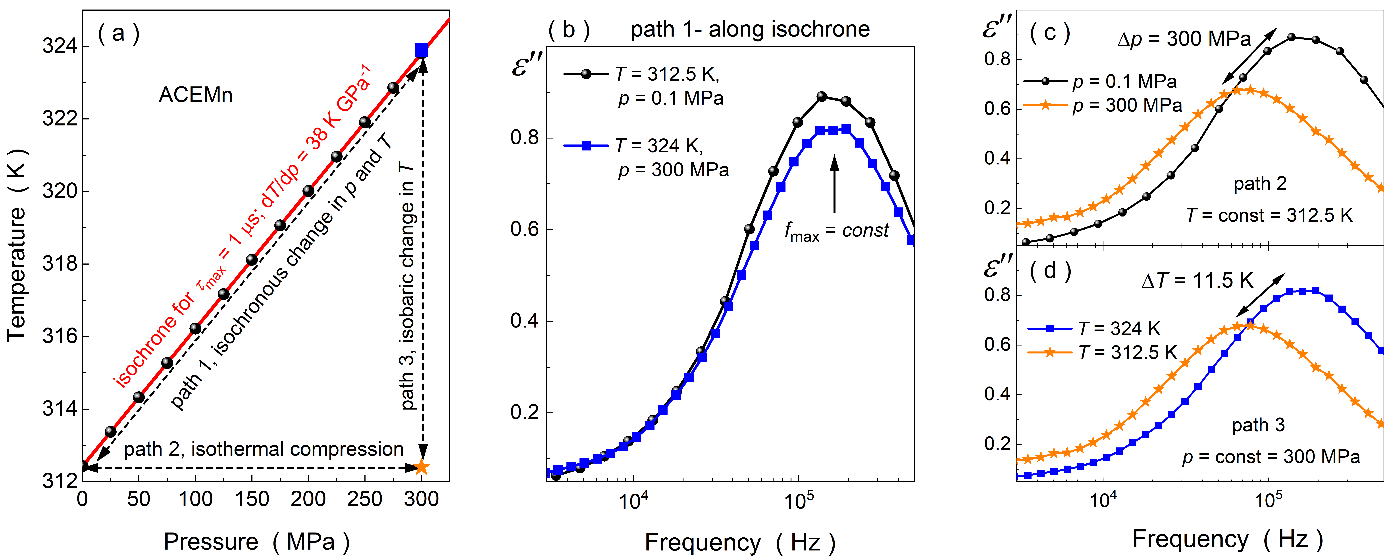
**

**Supplementary Figure 6. Relaxation dynamics in AceMn.** **(a)** Temperature-pressure conditions for AceMn required to keep the relaxation time constant and equal to 1 μs. Color points represents data calculated according to the equation of state, whereas red line is a linear fit of isochrone according to supplementary formula (8). **(b)** Comparison of relaxation peak position for AceMn between the ambient-pressure *ε"*(*f*) spectrum collected at 312.5 K and high-pressure *ε"*(*f*) spectrum collected at 324 K under 300 MPa. **(c)** Shift of relaxation peak at 312.5 K caused by the difference in pressure equal to 300 MPa. Experimental data collected for *p* = 0.1 MPa and *T* = 312.5 K are marked in black, whereas those obtained for *p* = 300 MPa and *T* = 312.5 K are marked in orange. **(d)** Shift of relaxation peak under 300 MPa caused by the difference in temperature equal to 11.5 K. Experimental data collected for *p* = 300 MPa and *T* = 324 K are marked in blue, whereas those obtained for *p* = 300 MPa and *T* = 312.5 K are marked in orange. Source data are provided as a Source Data file.

**
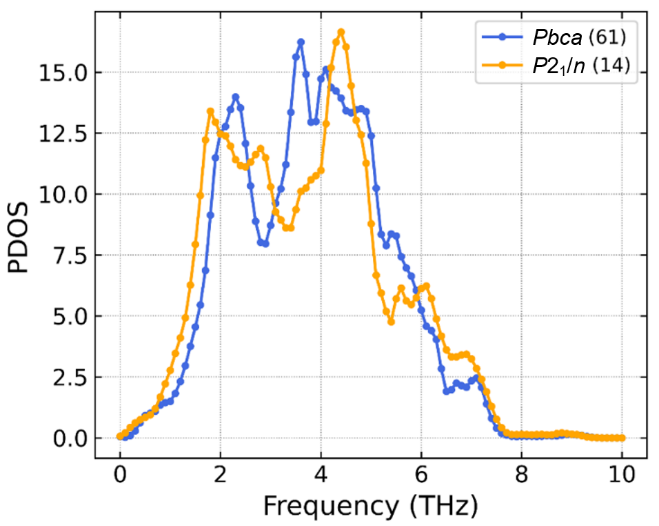
**

**Supplementary Figure 7. Phonon analysis for AceMn.** Phonon density of states (PDOS) projected on the atoms of acetamidine counterion for phases II and III in AceMn. The data in orange and blue are related to phases II and III, respectively.


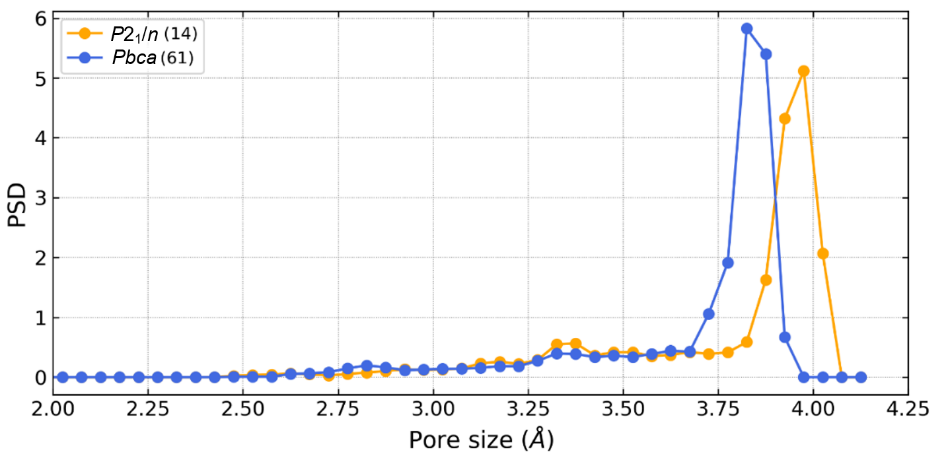


**Supplementary Figure 8. Pore size distribution (PSD) for two phases of AceMn (II and III).** The data in orange and blue are related to phases II and III, respectively. For the calculations, the counterion was removed from the framework and only void space within the pore was sampled.

**
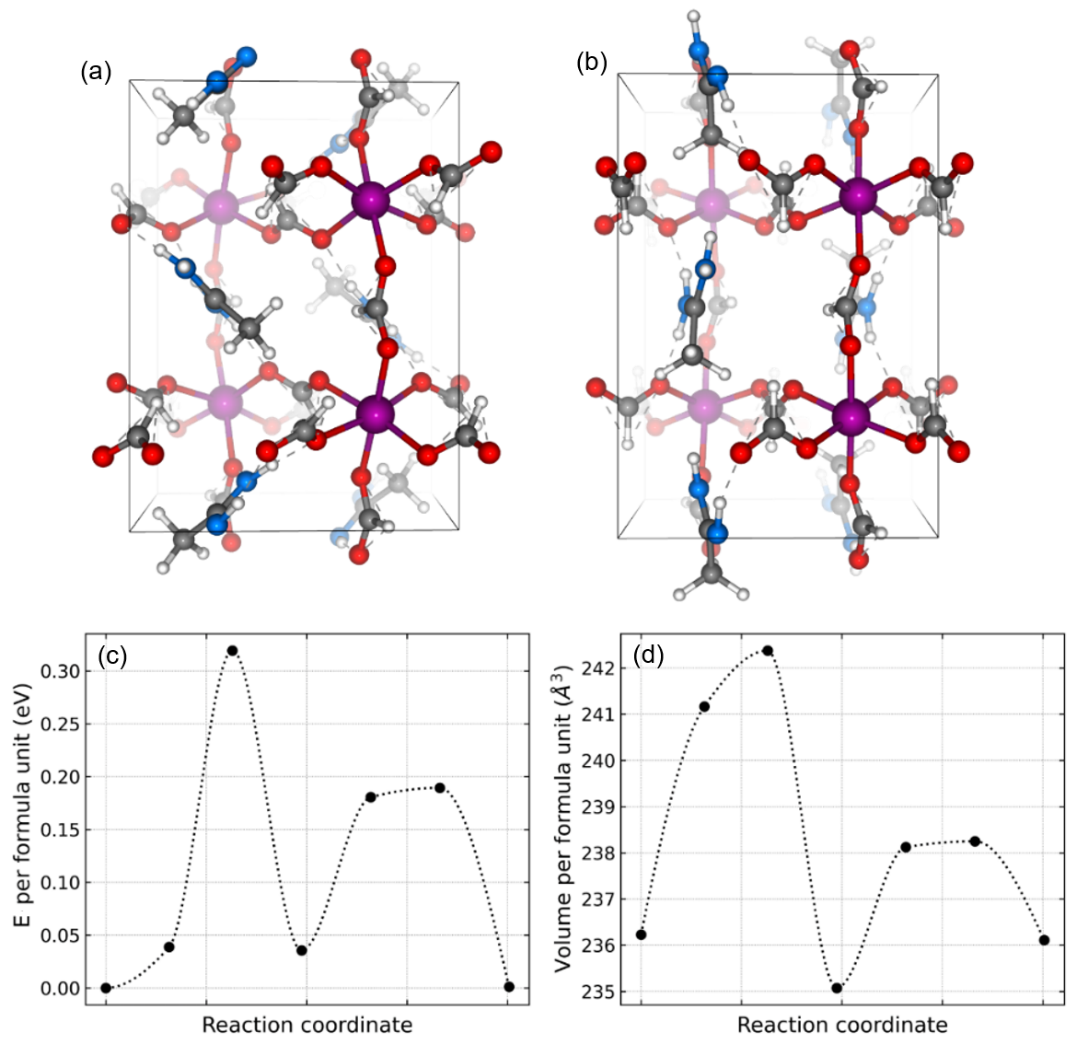
**

**Supplementary Figure 9. Structural consequences of the cage cation motion in AceMn.** (a) Arrangement of Ace^+^ cations within the metal-formate framework with minimum energy (0 on the reaction coordinate). Mn, O, C, N and H atoms are marked in purple, red, gray, blue, and white, respectively. (b) Transition state with the highest energy. Mn, O, C, N and H atoms are marked in purple, red, gray, blue, and white, respectively. (c) Changes in energy of the system during the rotation of Ace^+^ cations. (d) Variation of volume of the system (shown per formula unit) during the rotation of Ace+ cations.


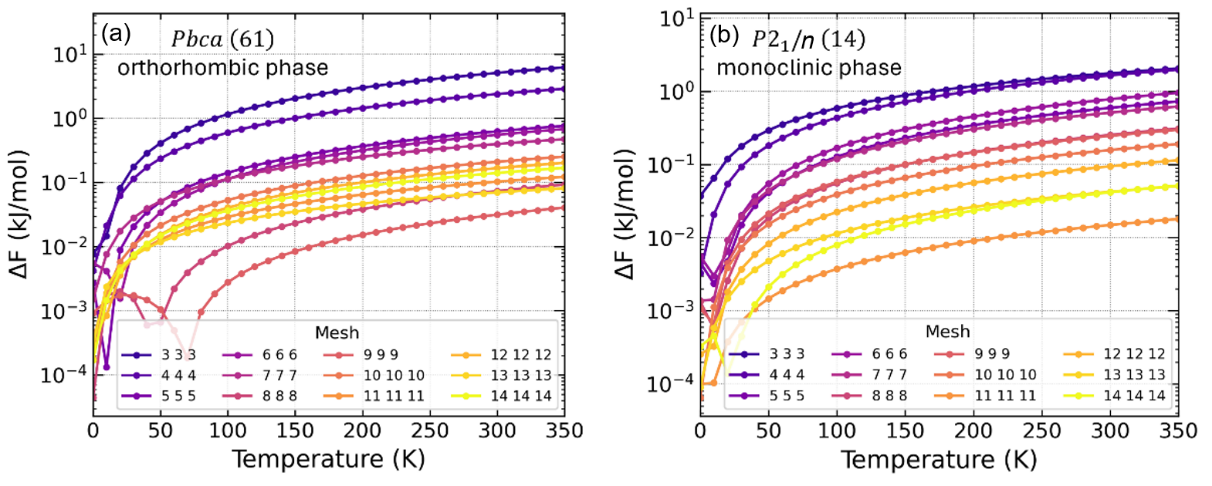


**Supplementary Figure 10.** **Free energy convergence.** Free energy convergence for different mesh grids used for phonon calculations with phonopy software for (a) orthorhombic and (b) monoclinic phases of AceMn. Each mesh grid is presented by separate color.

**Supplementary Notes**

**Supplementary Note 1**

The collected spectra of the complex dielectric permittivity were fitted in the frequency domain by means of the Havriliak-Negami function ^1^:

$\varepsilon^{*}\left( \omega\right)=\varepsilon_{\infty}+\frac{\Delta\varepsilon}{\left[ 1+\left( i\omega\tau_{\mathrm{HN}} \right)^{1-\alpha} \right]^{\beta}}$. (1)

In this formula *ε^*^* is the complex dielectric permittivity, *Δε* is dielectric strength, *ε*_∞­_ is the high-frequency limit of permittivity, *ω* is the angular frequency, *α* and *β* are the shape parameters and *τ* is the so-called Havriliak-Negami relaxation time, which is connected with the apparent relaxation time of the rotating cage cations by the shape parameters ^1^. Both real *ε'*(*f*) and imaginary *ε"*(*f*) parts of the complex dielectric permittivity were fitted by the same function simultaneously. The fitting procedure was conducted in the vicinity of the relaxation process exclusively, thereby allowing for the neglect of the contribution of conductivity contribution to the dielectric losses. This approach reduced the number of free parameters and had no influence on the accuracy of calculating the relaxation times because the relaxation peak maxima were well separated from the conductivity branch for both FMDMn and AceMn. Moreover, as exemplified by FMDMn, a satisfactory agreement between the fit function and experimental points was achieved for the relaxation process (see Supplementary Figure 5).

In this article, we will further focus and present only *ε"*(*f*) spectra because both real and imaginary parts of the complex dielectric permittivity are interrelated by Kramers-Kronig transform ^2^ and, thus, deliver exactly the same information in terms of relaxation phenomena:

$\varepsilon^{''}=-\frac{\pi}{2}\frac{\partial\varepsilon^{'}(\omega)}{\partial ln\omega}$ (2)

Besides, a detailed analysis of *ε'*(*f*) spectra has already been presented for these compounds^3–6^.

**Supplementary Note 2**

The pressure-temperature dependence of relaxation times in FMDMn and AceMn can be described with the following equation of state:

$\tau_{\max}(T, p)=\tau_{0}\exp\left( \frac{pV_{a}+E_{a,0MPa}}{RT} \right)$. (3)

In this formula, *V*_a_ is the activation volume, *E*_a,0MPa_ is the activation energy under 0 MPa, *R* is the gas constant and *τ*_0_ is the relaxation time at infinitely high *T* (when $\tau_{\max}\to\infty$) and *p* = 0 MPa. For a given isobaric conditions (*p* = *const*), this formula is reduced to a classical Arrhenius law, in which the activation energy is renormalized by pressure and *V*_a_ parameter:

$E_{a}\left( p \right)=E_{a (0MPa)}+pV_{a}$. (4)

Similarly, this equation of state can be reduced to a typical pressure form of Arrhenius law under isothermal conditions:

$\tau_{\max}(p)=\tau_{0.1MPa}\exp\left( \frac{pV_{a}}{RT} \right)$. (5)

In this formula, *τ*_0.1MPa_ is the ambient-pressure relaxation time at a given temperature, which can be calculated as:

$\tau_{0.1MPa}(T, p)=\tau_{0}\exp\left( \frac{E_{a}}{RT} \right)$. (6)

Hence, one can conclude that under isothermal conditions, the *τ_0_* parameter becomes renormalized instead of the activation energy *E*_a_.

Based on the equation of state (3), one can conclude that the activation volume *V*_a_ is a temperature-independent material constant that allows us to comprehend the whole high-pressure dielectric behaviour of 3D hybrid organic-inorganic perovskites. Specifically, it not only defines the volume requirements for the movement of A-site cage cations in these materials but also determines the:

(1) pressure-induced shift of the relaxation process and relaxation times according to equation (5),

(2) pressure-induced change in activation energy according to equation (4),

(3) pressure-temperature isochronous line of relaxation times.

To derive the mathematical formula for the last case, we rewrite the equation of state (3) to the following form:

$RT\ln\frac{\tau_{\max}}{\tau_{0}}=pV_{a}+E_{a,0MPa}$. (7)

Under isochronous conditions, the relaxation time *τ*_max_ remains constant, whereas *V*_a_, *τ*_0_ and *E*_a,0MPa_ are the material’s constants. That’s why one can write:

$T_{1}=\frac{p_{1}V_{a}}{R\ln\frac{\tau_{\max}}{\tau_{0}}}+\frac{E_{a,0MPa}}{R\ln\frac{\tau_{\max}}{\tau_{0}}}$; (8)

$T_{2}=\frac{p_{2}V_{a}}{R\ln\frac{\tau_{\max}}{\tau_{0}}}+\frac{E_{a,0MPa}}{R\ln\frac{\tau_{\max}}{\tau_{0}}}$. (9)

Finally, by subtracting these equations one gets:

$T_{1}-T_{2}=\left( p_{1}-p_{2} \right)\frac{V_{a}}{R\ln\frac{\tau_{\max(p)}}{\tau_{0}}}$; (10)

$\frac{\Delta T}{\Delta p}=\frac{dT}{dp}=\frac{V_{a}}{R\ln\frac{\tau_{\max(p)}}{\tau_{0}}}=const$ (11)

The last equation shows that the isochrone is described by a linear equation on the *p*-*T* surface for which the d*T*/d*p* coefficient adopts a constant value independently of selected pressure if the relaxation dynamics of a hybrid organic-inorganic compound is described by the equation of state (3). It also defines how to manipulate pressure and temperature to get the same relaxation time in such a system. For example, in the case of AceMn, the isochrone for *τ*_max_ = 1 μs is characterized by a d*T*/d*p* coefficient equal to 38 K GPa^-1^. It means that one needs to elevate the temperature by 38 K to keep the same relaxation time when the pressure increases by 1 GPa. Equivalently, the raise in temperature by 11.4 K is needed when pressure increases by 300 MPa (see Supplementary Figure 6a and 6b). It also means that the application of external hydrostatic pressure of 300 MPa exerts the same effect on relaxation times as the temperature decrease by merely 11.4 K (see Supplementary Figure 6c and 6d). Hence, the is d*T*/d*p* coefficient a powerful parameter to estimate which thermodynamic variable (pressure, temperature) exerts a more significant impact on relaxation dynamics in hybrid organic-inorganic compounds.

**Supplementary Tables**

**Supplementary Table 1.** Crystallographic data of AceMn at 130, 160, 200, 240 and 280 K under ambient pressure.

| CSD Number | | 2287917 | 2287918 | 2287919 | 2287920 | 2287921 |
| --- | --- | --- | --- | --- | --- | --- |
| Pressure (GPa) | | 0.0001 | 0.0001 | 0.0001 | 0.0001 | 0.0001 |
| Temperature (K) | | 130 | 160 | 200 | 240 | 280 |
| Formula weight | | 249.09 | 249.09 | 249.09 | 249.09 | 249.09 |
| Wavelength (Å) | | 1.54184 | 1.54184 | 1.54184 | 1.54184 | 1.54184 |
| Crystal system | | Monoclinic | Monoclinic | Monoclinic | Monoclinic | Monoclinic |
| Space group | | *P*2_1_/*n* | *P*2_1_/*n* | *P*2_1_/*n* | *P*2_1_/*n* | *P*2_1_/*n* |
| Unit cell dimensions (Å,°) | *a* | 8.6907(3) | 8.6988(4) | 8.7126(5) | 8.7246(4) | 8.7461(5) |
|  | *b* | 11.9475(4) | 11.9620(4) | 11.9886(6) | 12.0134(5) | 12.0467(6) |
|  | *c* | 9.0389(4) | 9.0360(4) | 9.0330(5) | 9.0303(4) | 9.0307(5) |
|  | *β* | 92.368(4) | 92.298(4) | 92.083(5) | 91.820(5) | 91.061(5) |
| Volume (Å^3^) | | 937.73(6) | 939.48(7) | 942.89(9) | 946.01(7) | 951.33(9) |
| *Z/Z’* | | 4/1 | 4/1 | 4/1 | 4/1 | 4/1 |
| Calculated density (gcm^-3^) | | 1.764 | 1.761 | 1.755 | 1.749 | 1.739 |
| Absorption coefficient (mm^-1^) | | 11.614 | 11.592 | 11.550 | 11.512 | 11.448 |
| F(000) | | 508 | 508 | 508 | 508 | 508 |
| Crystal size (mm) | | 0.1·0.08·0.05 | 0.1·0.08·0.05 | 0.1·0.08·0.05 | 0.1·0.08·0.05 | 0.1·0.08·0.05 |
| Θ-range for data collection (°) | | 6.1130 to 72.3030 | 6.0700 to 71.5190 | 6.1140 to 71.6370 | 6.1160 to 71.6250 | 6.0630 to 71.8750 |
| Min/max indices: *h*, *k*, *l* | | -10/10,  -14/10, -8/11 | -10/10,  -14/10, -8/11 | -10/10,  -14/10, -8/11 | -10/10,  -14/10, -8/11 | -10/10,  -14/10, -8/11 |
| Reflect. Collected/unique | | 3839/1692 | 3682/1679 | 3748/1697 | 3691/1698 | 3775/1703 |
| *R*_int_/ Completeness (%) | | 0.0291/98.6 | 0.0292/98.8 | 0.0321/98.3 | 0.0349/99.0 | 0.0300/98.7 |
| Data/restrains/parameters | | 1692/0/128 | 1679/0/128 | 1697/0/128 | 1698/0/128 | 1703/0/128 |
| Goodness-of-fit on *F*^2^ | | 1.084 | 1.105 | 1.048 | 1.075 | 1.086 |
| Final *R*_1_/w*R*_2_ (*I*>2*δ*_1_) | | 0.0610/0.1839 | 0.0606/0.1874 | 0.0647/0.1899 | 0.0683/0.2093 | 0.0858/0.2491 |
| *R*_1_/w*R*^2^ (all data) | | 0.0685/0.1959 | 0.0689/0.2004 | 0.0741/0.2031 | 0.0792/0.2264 | 0.0977/0.2689 |

**Supplementary Table 2.** Ambient-pressure crystallographic data of AceMn obtained at 296, 300, 302, 305 K, as well as similar room-temperature high-pressure data collected under 0.45 GPa.

| CSD Number | | 2287922 | 2287923 | 2287924 | 2287925 | 2287926 |
| --- | --- | --- | --- | --- | --- | --- |
| Pressure (GPa) | | 0.0001 | 0.0001 | 0.0001 | 0.0001 | 0.45 |
| Temperature (K) | | 296 | 300 | 302 | 305 | 293 |
| Formula weight | | 249.09 | 249.09 | 249.09 | 249.09 | 249.09 |
| Wavelength (Å) | | 1.54184 | 1.54184 | 1.54184 | 1.54184 | 0.71074 |
| Crystal system | | Monoclinic | Orthorhombic | Orthorhombic | Orthorhombic | Monoclinic |
| Space group | | *P*2_1_/*n* | *Imma* | *Imma* | *Imma* | *P*2_1_/*n* |
| Unit cell dimensions (Å,°) | *a* | 8.7440(4) | 8.7433(3) | 8.7422(3) | 8.7417(3) | 8.640(7) |
|  | *b* | 12.0563(4) | 12.0592(4) | 12.0626(3) | 12.0648(3) | 11.920(3) |
|  | *c* | 9.0175(4) | 9.0164(3) | 9.0168(3) | 9.0167(3) | 9.031(2) |
|  | *β* | 90.099(5) | 90 | 90 | 90 | 91.38(4) |
| Volume (Å^3^) | | 950.63(7) | 950.66(6) | 950.85(5) | 950.96(5) | 929.9(8) |
| *Z/Z’* | | 4/1 | 4/1 | 4/1 | 4/1 | 4/1 |
| Calculated density  (gcm^-3^) | | 1.740 | 1.740 | 1.740 | 1.740 | 1.779 |
| Absorption coefficient (mm^-1^) | | 11.456 | 11.456 | 11.453 | 11.452 | 1.427 |
| F(000) | | 508 | 508 | 508 | 508 | 508 |
| Crystal size (mm) | | 0.1·0.08·0.05 | 0.1·0.08·0.05 | 0.1·0.08·0.05 | 0.1·0.08·0.05 | 0.2·0.2·0.05 |
| Θ-range for data collection (°) | | 6.0930 to 72.2040 | 6.1050 to 72.2530 | 6.1020 to 72.2700 | 6.1020 to 72.2600 | 3.6170 to 26.4870 |
| Min/max indices: *h*, *k*, *l* | | -10/10,  -14/10, -8/11 | -10/10,  -14/10, -8/11 | -10/10,  -14/10, -8/11 | -10/10,  -14/10, -7/11 | -7/6, -14/14,  -11/11 |
| Reflect. Collected/unique | | 3933/1721 | 1903/523 | 1904/522 | 1887/521 | 2946/738 |
| *R*_int_/ Completeness (%) | | 0.0187/98.6 | 0.0146/99.2 | 0.0173/99.0 | 0.0148/98.6 | 0.1241/54.6 |
| Data/restrains/ parameters | | 1721/0/128 | 523/4/64 | 522/5/64 | 521/13/64 | 738/48/129 |
| Goodness-of-fit on *F*^2^ | | 1.082 | 1.054 | 1.089 | 1.066 | 0.993 |
| Final *R*_1_/w*R*_2_ (*I*>2*δ*_1_) | | 0.0382/0.1038 | 0.0258/0.0690 | 0.0260/0.0698 | 0.0256/0.0691 | 0.0720/0.1797 |
| *R*_1_/w*R*^2^ (all data) | | 0.0503/0.1142 | 0.0277/0.0711 | 0.0275/0.0716 | 0.0273/0.0711 | 0.1292/0.2158 |

**Supplementary Table 3.** Detailed high-pressure crystallographic data of AceMn obtained at room temperature under 0.86, 0.95, 1.34, 1.60 and 2.10 GPa.

| CSD Number | | 2287927 | 2287928 | 2287929 | 2287930 | 2287931 |
| --- | --- | --- | --- | --- | --- | --- |
| Pressure (GPa) | | 0.86 | 0.95 | 1.34 | 1.60 | 2.10 |
| Temperature (K) | | 293 | 293 | 293 | 293 | 293 |
| Formula weight | | 249.09 | 249.09 | 249.09 | 249.09 | 249.09 |
| Wavelength (Å) | | 0.71074 | 0.71074 | 0.71074 | 0.71074 | 0.71074 |
| Crystal system | | Monoclinic | Orthorhombic | Orthorhombic | Orthorhombic | Orthorhombic |
| Space group | | *P*2_1_/*n* | *Pbca* | *Pbca* | *Pbca* | *Pbca* |
| Unit cell dimensions (Å,°) | *a* | 8.590(7) | 11.894(4) | 11.888(8) | 11.771(6) | 11.737(8) |
|  | *b* | 11.862(3) | 11.68(3) | 11.674(3) | 11.592(2) | 11.488(3) |
|  | *c* | 9.051(2) | 12.905(3) | 12.819(3) | 12.803(3) | 12.751(3) |
|  | *β* | 91.81(5) | 90 | 90 | 90 | 90 |
| Volume (Å^3^) | | 921.8(8) | 1793(4) | 1779.1(13) | 1747.0(11) | 1719.2(13) |
| *Z/Z’* | | 4/1 | 8/1 | 8/1 | 8/1 | 8/1 |
| Calculated density (gcm^-3^) | | 1.795 | 1.846 | 1.860 | 1.894 | 1.925 |
| Absorption coefficient  (mm^-1^) | | 1.440 | 1.481 | 1.492 | 1.519 | 1.544 |
| F(000) | | 508 | 1016 | 1016 | 1016 | 1016 |
| Crystal size (mm) | | 0.2·0.2·0.05 | 0.22·0.2·0.05 | 0.2·0.2·0.05 | 0.2·0.2·0.05 | 0.2·0.2·0.05 |
| Θ-range for data collection (°) | | 4.0890 to 23.4110 | 3.0850 to 19.3910 | 3.5130 to 23.8380 | 3.5450 to 23.9490 | 3.6030 to 22.8320 |
| Min/max indices: *h*, *k*, *l* | | -7/7, -14/14, -11/11 | -3/3, -10/10, -12/12 | -8/8, -14/14, -15/15 | -8/8, -13/14, -15/16 | -8/8, -13/15, -14/15 |
| Reflect. Collected/unique | | 3396/776 | 14855/239 | 5215/1039 | 4540/1042 | 5054/997 |
| *R*_int_/ Completeness (%) | | 0.1498/41.1 | 0.2329/30.1 | 0.1564/72.5 | 0.2009/72.7 | 0.2360/71.0 |
| Data/restrains/parameters | | 776/42/129 | 239/1/58 | 1039/150/129 | 1042/144/129 | 997/123/128 |
| Goodness-of-fit on *F*^2^ | | 0.990 | 1.089 | 1.035 | 0.985 | 1.034 |
| Final *R*_1_/w*R*_2_ (*I*>2*δ*_1_) | | 0.0749/0.1761 | 0.0915/0.1798 | 0.0885/0.2065 | 0.0899/0.1989 | 0.0906/0.2165 |
| *R*_1_/w*R*^2^ (all data) | | 0.1351/0.2113 | 0.1630/0.2303 | 0.1570/0.2579 | 0.1707/0.2532 | 0.1714/0.2786 |

**Supplementary Table 4.** Detailed high-pressure crystallographic data of AceMn obtained at room temperature under 2.58, 2.90, 3.03, 3.39 and 4.10 GPa.

| CSD Number | | 2287932 | 2287933 | 2287934 | 2287935 | 2287936 |
| --- | --- | --- | --- | --- | --- | --- |
| Pressure (GPa) | | 2.58 | 2.90 | 3.03 | 3.39 | 4.10 |
| Temperature (K) | | 293 | 293 | 293 | 293 | 293 |
| Formula weight | | 249.09 | 249.09 | 249.09 | 249.09 | 249.09 |
| Wavelength (Å) | | 0.71074 | 0.71074 | 0.71074 | 0.71074 | 0.71074 |
| Crystal system | | Orthorhombic | Orthorhombic | Orthorhombic | Orthorhombic | Orthorhombic |
| Space group | | *Pbca* | *Pbca* | *Pbca* | *Pbca* | *Pbca* |
| Unit cell dimensions (Å,°) | *a* | 11.674(8) | 11.616(9) | 11.56(3) | 11.493(3) | 11.356(13) |
|  | *b* | 11.378(2) | 11.321(3) | 11.232(4) | 11.304(8) | 11.165(4) |
|  | *c* | 12.658(3) | 12.669(4) | 12.609(3) | 12.649(7) | 12.528(16) |
|  | *β* | 90 | 90 | 90 | 90 | 90 |
| Volume (Å^3^) | | 1681.3(12) | 1666.0(15) | 1638(4) | 1643.4(15) | 1588(3) |
| *Z/Z’* | | 8/1 | 8/1 | 8/1 | 8/1 | 8/1 |
| Calculated density (gcm^-3^) | | 1.968 | 1.986 | 2.021 | 2.014 | 2.083 |
| Absorption coefficient (mm^-1^) | | 1.579 | 1.593 | 1.621 | 1.615 | 1.671 |
| F(000) | | 1016 | 1016 | 1016 | 1016 | 1016 |
| Crystal size (mm) | | 0.2·0.2·0.05 | 0.2·0.2·0.050 | 0.22·0.2·0.05 | 0.2·0.2·0.05 | 0.2·0.2·0.05 |
| Θ-range for data collection (°) | | 3.6110 to 22.0610 | 3.6310 to 25.9900 | 3.1910 to 19.6730 | 3.5950 to 24.1530 | 3.5690 to 22.1460 |
| Min/max indices: *h*, *k*, *l* | | -8/8, -13/13, -14/15 | -8/8, -13/13, -14/15 | -2/2, -11/11, -12/13 | -7/6, -10/11, -10/9 | -7/6, -10/11, -10/9 |
| Reflect. Collected/unique | | 6901/1039 | 3998/880 | 14916/226 | 805/386 | 814/388 |
| *R*_int_/ Completeness (%) | | 0.1966/71.3 | 0.1957/65.5 | 0.3917/22.8 | 0.1116/60.4 | 0.1121/61.4 |
| Data/restrains/ parameters | | 1039/144/128 | 880/174/129 | 226/80/128 | 386/138/129 | 388/138/129 |
| Goodness-of-fit on *F*^2^ | | 1.034 | 0.984 | 1.198 | 1.029 | 1.050 |
| Final *R*_1_/w*R*_2_ (*I*>2*δ*_1_) | | 0.0857/0.2063 | 0.0962/0.2167 | 0.0508/0.0798 | 0.0706/0.1241 | 0.0732/0.1292 |
| *R*_1_/w*R*^2^ (all data) | | 0.1644/0.2610 | 0.2141/0.2960 | 0.1459/0.1367 | 0.1781/0.1750 | 0.1791/0.1746 |

**Supplementary Table 5.** Detailed high-pressure crystallographic data of AceMn obtained at room temperature under 5.20, 5.40, 6.00 and 7.35 GPa.

| CSD Number | | 2287937 | 2287938 | 2287939 | 2287940 |
| --- | --- | --- | --- | --- | --- |
| Pressure (GPa) | | 5.20 | 5.40 | 6.00 | 7.35 |
| Temperature (K) | | 293 | 293 | 293 | 293 |
| Formula weight | | 249.09 | 249.09 | 249.09 | 249.09 |
| Wavelength (Å) | | 0.71074 | 0.71074 | 0.71074 | 0.71074 |
| Crystal system | | Orthorhombic | Orthorhombic | Orthorhombic | Orthorhombic |
| Space group | | *Pbca* | *Pbca* | *Pbca* | *Pbca* |
| Unit cell dimensions (Å,°) | *a* | 11.356(15) | 11.384(18) | 11.221(19) | 11.07(2) |
|  | *b* | 10.9633(15) | 10.9105(17) | 10.9223(18) | 10.919(2) |
|  | *c* | 12.4937(13) | 12.4299(17) | 12.4475(18) | 12.461(2) |
|  | *β* | 90 | 90 | 90 | 90 |
| Volume (Å^3^) | | 1555(2) | 1544(2) | 1526(3) | 1506(3) |
| *Z/Z’* | | 8/1 | 8/1 | 8/1 | 8/1 |
| Calculated density (gcm^-3^) | | 2.127 | 2.143 | 2.169 | 2.197 |
| Absorption coefficient  (mm^-1^) | | 1.706 | 1.719 | 1.740 | 1.762 |
| F(000) | | 1016 | 1016 | 1016 | 1016 |
| Crystal size (mm) | | 0.22·0.2·0.05 | 0.22·0.2·0.05 | 0.22·0.2·0.05 | 0.22·0.2·0.05 |
| Θ-range for data collection (°) | | 3.1800 to 18.6160 | 3.1840 to 20.9780 | 2.0490 to 18.6010 | 3.2070 to 18.6660 |
| Min/max indices: *h*, *k*, *l* | | -2/2, -10/10, -12/12 | -2/3, -10/11, -12/13 | -2/2, -10/10, -12/12 | -3/3, -10/10, -12/12 |
| Reflect. Collected/unique | | 11621/200 | 29333/240 | 28216/242 | 14665/236 |
| *R*_int_/ Completeness (%) | | 0.2170/29.4 | 0.3487/24.8 | 0.3111/29.4 | 0.2308/29.7 |
| Data/restrains/parameters | | 200/28/63 | 240/7/63 | 242/3/58 | 236/2/58 |
| Goodness-of-fit on *F*^2^ | | 1.146 | 1.183 | 1.058 | 1.082 |
| Final *R*_1_/w*R*_2_ (*I*>2*δ*_1_) | | 0.0996/0.2186 | 0.0980/0.2484 | 0.0974/0.2140 | 0.0842/0.1886 |
| *R*_1_/w*R*^2^ (all data) | | 0.1698/0.2842 | 0.1665/0.2947 | 0.1843/0.2804 | 0.1524/0.2518 |

**Supplementary Table 6.** Detailed room-temperature crystallographic data of FMDMn obtained under 0.10 MPa, as well as 0.16, 0.35, 0.56 and 0.85 GPa.

| CSD Number | | 2287942 | 2287943 | 2287944 | 2287945 | 2287946 |
| --- | --- | --- | --- | --- | --- | --- |
| Pressure (GPa) | | 0.0001 | 0.16 | 0.35 | 0.56 | 0.85 |
| Temperature (K) | | 293 | 293 | 293 | 293 | 293 |
| Formula weight | | 235.06 | 235.06 | 235.06 | 235.06 | 235.06 |
| Wavelength (Å) | | 0.71074 | 0.71074 | 0.71074 | 0.71074 | 0.71074 |
| Crystal system | | Monoclinic | Monoclinic | Monoclinic | Monoclinic | Monoclinic |
| Space group | | *C*2/*c* | *C*2/*c* | *C*2/*c* | *C*2/*c* | *C*2/*c* |
| Unit cell dimensions (Å,°) | *a* | 13.8051(9) | 13.627(4) | 13.48(3) | 13.30(2) | 13.107(19) |
|  | *b* | 8.6947(3) | 8.681(2) | 8.6608(7) | 8.6579(6) | 8.6423(6) |
|  | *c* | 8.4685(5) | 8.441(6) | 8.392(8) | 8.358(7) | 8.299(5) |
|  | *β* | 119.715(8) | 119.223(18) | 118.39(19) | 117.83(16) | 116.94(13) |
| Volume (Å^3^) | | 882.82(10) | 871.5(7) | 862(2) | 851(2) | 838.1(16) |
| *Z/Z’* | | 4/1 | 4/1 | 4/1 | 4/1 | 4/1 |
| Calculated density  (gcm^-3^) | | 1.769 | 1.792 | 1.811 | 1.834 | 1.863 |
| Absorption coefficient  (mm^-1^) | | 1.498 | 1.517 | 1.534 | 1.553 | 1.578 |
| F(000) | | 476 | 476 | 476 | 476 | 476 |
| Crystal size (mm) | | 0.18·0.12·0.06 | 0.22·0.2·0.05 | 0.22·0.2·0.05 | 0.22·0.2·0.05 | 0.22·0.2·0.05 |
| Θ-range for data collection (°) | | 4.5390 to 25.6050 | 3.3980 to 23.1110 | 4.6990 to 24.5660 | 4.6970 to 24.4810 | 4.7090 to 24.5130 |
| Min/max indices: *h*, *k*, *l* | | -18/18,  -11/11, -11/11 | -11/11,  -11/11, -11/10 | -10/10,  -10/10, -10/10 | -10/10,  -10/10, -10/10 | -9/9, -10/10,  -10/10 |
| Reflect. Collected/unique | | 13347/1117 | 1591/279 | 2046/247 | 2043/248 | 2047/251 |
| *R*_int_/ Completeness (%) | | 0.0558/99.9 | 0.1636/39.6 | 0.0450/30.2 | 0.0449/36.4 | 0.0455/31.3 |
| Data/restrains/ parameters | | 1117/4/71 | 279/134/71 | 247/64/71 | 248/86/71 | 251/74/71 |
| Goodness-of-fit on *F*^2^ | | 1.084 | 1.077 | 1.087 | 1.198 | 1.127 |
| Final *R*_1_/w*R*_2_ (*I*>2*δ*_1_) | | 0.0398/0.0974 | 0.0717/0.1744 | 0.0259/0.0657 | 0.0367/0.0984 | 0.0362/0.1048 |
| *R*_1_/w*R*^2^ (all data) | | 0.0557/0.1050 | 0.1026/0.1942 | 0.0362/0.0720 | 0.0472/0.1060 | 0.0456/0.1110 |

**Supplementary Table 7.** Detailed high-pressure crystallographic data of FMDMn obtained at room temperature under 1.09, 1.54, 1.95, 2.35 and 2.51 GPa.

| CSD Number | | 2287947 | 2287948 | 2287949 | 2287950 | 2287951 |
| --- | --- | --- | --- | --- | --- | --- |
| Pressure (GPa) | | 1.09 | 1.54 | 1.95 | 2.35 | 2.51 |
| Temperature (K) | | 293 | 293 | 293 | 293 | 293 |
| Formula weight | | 235.06 | 235.06 | 235.06 | 235.06 | 235.06 |
| Wavelength (Å) | | 0.71074 | 0.71074 | 0.71074 | 0.71074 | 0.71074 |
| Crystal system | | Monoclinic | Monoclinic | Monoclinic | Monoclinic | Monoclinic |
| Space group | | *C*2/*c* | *C*2/*c* | *C*2/*c* | *C*2/*c* | *C*2/*c* |
| Unit cell dimensions (Å,°) | *a* | 12.92(2) | 12.60(2) | 12.428(18) | 12.28(2) | 12.1953(17) |
|  | *b* | 8.6313(6) | 8.6301(6) | 8.6177(6) | 8.6166(12) | 8.6125(19) |
|  | *c* | 8.237(6) | 8.137(5) | 8.064(4) | 8.006(9) | 7.9769(18) |
|  | *β* | 116.14(14) | 114.95(13) | 114.22(12) | 113.6(2) | 113.20(2) |
| Volume (Å^3^) | | 824.6(17) | 802.1(16) | 787.6(14) | 776(2) | 770.1(3) |
| *Z/Z’* | | 4/1 | 4/1 | 4/1 | 4/1 | 4/1 |
| Calculated density  (gcm^-3^) | | 1.893 | 1.947 | 1.982 | 2.011 | 2.027 |
| Absorption coefficient  (mm^-1^) | | 1.603 | 1.648 | 1.679 | 1.703 | 1.717 |
| F(000) | | 476 | 476 | 476 | 476 | 476 |
| Crystal size (mm) | | 0.22·0.2·0.05 | 0.22·0.2·0.05 | 0.22·0.2·0.05 | 0.22·0.2·0.05 | 0.22·0.2·0.05 |
| Θ-range for data collection (°) | | 4.7120 to 24.5310 | 4.7130 to 24.9470 | 4.7190 to 24.9730 | 4.6780 to 24.5340 | 4.5330 to 23.2950 |
| Min/max indices: *h*, *k*, *l* | | -9/9, -10/10, -10/10 | -9/9, -10/10, -10/10 | -8/8, -10/10, -10/10 | -8/8, -10/10, -10/10 | -14/14, -8/8, -7/7 |
| Reflect. Collected/unique | | 1980/243 | 1929/235 | 1863/229 | 1841/237 | 1845/325 |
| *R*_int_/ Completeness (%) | | 0.0448/31.2 | 0.0422/30.6 | 0.0392/30.2 | 0.0520/38.1 | 0.0295/43.5 |
| Data/restrains/ parameters | | 243/50/62 | 235/52/62 | 229/56/63 | 237/62/62 | 325/7/62 |
| Goodness-of-fit on *F*^2^ | | 1.108 | 1.128 | 1.091 | 1.127 | 1.136 |
| Final *R*_1_/w*R*_2_ (*I*>2*δ*_1_) | | 0.0304/0.0800 | 0.0289/0.0704 | 0.0260/0.0648 | 0.0321/0.0692 | 0.0503/0.1273 |
| *R*_1_/w*R*^2^ (all data) | | 0.0377/0.0844 | 0.0361/0.0737 | 0.0360/0.0689 | 0.0515/0.0775 | 0.0653/0.1393 |

**Supplementary Table 8.** High-pressure crystallographic data of FMDMn obtained at room-temperature under 3.04 and 3.63 GPa.

| CSD Number | | 2287952 | 2287953 |
| --- | --- | --- | --- |
| Pressure (GPa) | | 3.04 | 3.63 |
| Temperature (K) | | 293 | 293 |
| Formula weight | | 235.06 | 235.06 |
| Wavelength (Å) | | 0.71074 | 0.71074 |
| Crystal system | | Monoclinic | Monoclinic |
| Space group | | *C*2/*c* | *C*2/*c* |
| Unit cell dimensions (Å,°) | *a* | 12.0212(19) | 11.894(2) |
|  | *b* | 8.601(2) | 8.588(2) |
|  | *c* | 7.928(2) | 7.902(2) |
|  | *β* | 112.77(2) | 112.54(3) |
| Volume (Å^3^) | | 755.8(3) | 745.5(4) |
| *Z/Z’* | | 4/1 | 4/1 |
| Calculated density (gcm^-3^) | | 2.066 | 2.094 |
| Absorption coefficient (mm^-1^) | | 1.749 | 1.773 |
| F(000) | | 476 | 476 |
| Crystal size (mm) | | 0.22·0.2·0.05 | 0.22·0.2·0.05 |
| Θ-range for data collection (°) | | 4.5300 to 23.3460 | 4.5670 to 23.4300 |
| Min/max indices: *h*, *k*, *l* | | -13/13, -8/8,  -7/7 | -13/13, -8/8,  -7/7 |
| Reflect. Collected/unique | | 1666/296 | 1659/299 |
| *R*_int_/ Completeness (%) | | 0.0354/40.6 | 0.0380/42.0 |
| Data/restrains/parameters | | 296/8/62 | 299/8/62 |
| Goodness-of-fit on *F*^2^ | | 1.157 | 1.127 |
| Final *R*_1_/w*R*_2_ (*I*>2*δ*_1_) | | 0.0452/0.1135 | 0.0426/0.1045 |
| *R*_1_/w*R*^2^ (all data) | | 0.0551/0.1199 | 0.0616/0.1156 |

**Supplementary Table 9.** Compressibility related to crystallographic axes calculated for the phase II calculated in the range between 0.1 MPa and 3.63 GPa for FMDMn.

| Axes | *K*(TPa^-1^) | *σK*(TPa^-1^) | Direction | | | Empirical parameters | | | |
| --- | --- | --- | --- | --- | --- | --- | --- | --- | --- |
|  |  |  | *a* | *b* | *c* | *ε*_0_ | *λ* | *P*_c_ | *ν* |
| X_1_ | 50.3566 | 1.6481 | 0.8679 | 0.0 | -0.4968 | 212.829 | -212.7965 | -1.2906 | 0.0006 |
| X_2_ | 3.0584 | 0.2162 | -0.0 | 1.0 | -0.0 | 0.0012 | -0.0073 | -0.017 | 0.4425 |
| X_3_ | -4.197 | 0.4866 | 0.4045 | 0.0 | 0.9145 | -39.7359 | 39.7462 | -0.1151 | 0.0001 |
| V | 44.0792 | 2.7196 |  |  |  |  |  |  |  |

**Supplementary Table 10.** Birch-Murnaghan Coefficients for FMDMn.

|  | *B*_0_ (GPa) | σ*B*_0_ (GPa) | *V*_0_ (Å^3^) | *σV*_0_ (Å^3^) | [*B*'](http://pascal.chem.ox.ac.uk/cgi-bin/first.cgi) | [*σB*'](http://pascal.chem.ox.ac.uk/cgi-bin/first.cgi) | [*P*_c_ (GPa)](http://pascal.chem.ox.ac.uk/cgi-bin/first.cgi) |
| --- | --- | --- | --- | --- | --- | --- | --- |
| 2nd | 14.9322 | 0.5066 | 879.9153 | 2.6754 | 4.0 | n/a | 0.0 |
| 3rd | 10.7024 | 1.5327 | 887.0257 | 4.1571 | 7.8548 | 1.6858 | 0.0 |

**Supplementary Table 11.** Wavenumber intercepts at zero pressure (*ω*_0_) and pressure coefficients (*α*=d*ω*/d*p*), obtained from fitting of the experimental data by linear functions, for the phases II, III, and IV of FMDMn.

| Ambient-pressure phase II | | High-pressure phase III | | High-pressure phase IV | | Assignement* |
| --- | --- | --- | --- | --- | --- | --- |
| *ω*_0_  (cm^-1^) | *α*  (cm^-1^GPa^-1^) | *ω*_0_  (cm^-1^) | *α*  (cm^-1^GPa^-1^) | *ω*_0_  (cm^-1^) | *α*  (cm^-1^GPa^-1^) |  |
| 1440.0 | 3.20 | 1437.9 | 3.38 | 1454.7 | 2.15 | *δ*(CH)+*ν*_as_(CN) |
| 1429.7 | 1.34 | 1427.7 | 1.86 | 1428.0 | 1.65 | *δ*(CH) |
| 1380.8 | 5.55 | 1388.2 | 1.88 | 1384.0 | 3.38 | *ν*_5_(HCOO^-^) |
| 1374.2 | 3.16 | 1370.5 | 4.74 | 1380.8 | 2.64 | *ν*_5_(HCOO^-^) |
| 1371.6 | 2.21 | 1367.8 | 3.09 | 1363.3 | 3.44 | *ν*_5_(HCOO^-^) |
| 1364.4 | 3.31 | 1365.9 | 2.13 | 1360.3 | 2.99 | *ν*_2_(HCOO^-^) |
|  |  | 1346.1 | 3.94 | 1370.1 | 0.89 | *ν*_2_(HCOO^-^) |
| 1358.4 | 2.99 | 1366.5 | 0.32 | 1363.2 | 0.72 | *ν*_2_(HCOO^-^) |
| 1159.3 | -5.84 | 1127.4 | 4.36 | 1134.5 | 3.07 | *ν*_s_(CN) |
| 1145.3 | -4.81 | 1134.9 | -1.12 | 1125.8 | 0.29 | *ν*_s_(CN) |
| 1061.7 | 3.18 | 1071.7 | 1.31 | 1082.8 | -0.29 | *ν*_6_(HCOO^-^) |
| 793.9 | 1.17 | 790.2 | 1.73 | 780.4 | 2.80 | *ν*_3_(HCOO^-^) |
|  |  |  |  | 784.7 | -1.96 | *ν*_3_(HCOO^-^) |
| 590.7 | 1.12 | 587.1 | 2.02 | 562.3 | 4.71 | *δ*(CN)+*τ*(NH_2_) |
|  |  | 588.2 | -0.50 | 591.4 | -0.99 | *δ*(CN)+*τ*(NH_2_) |
|  |  |  |  | 593.3 | -3.50 | *δ*(CN)+*τ*(NH_2_) |
| 258.8 | 18.16 | 261.2 | 16.72 | 289.5 | 13.12 | *T’*(Mn^2+^)+*T’*(HCOO^-^) |
|  |  | 243.0 | 13.35 | 234.1 | 14.46 | *T’*(Mn^2+^)+*T’*(HCOO^-^) |
| 237.6 | 10.60 | 239.1 | 9.14 | 199.6 | 14.47 | *T’*(Mn^2+^)+*T’*(HCOO^-^) |
| 192.1 | 11.62 | 205.2 | 9.25 | 207.1 | 9.41 | *T’*(Mn^2+^)+*T’*(HCOO^-^) |
| 165.0 | 10.74 | 177.0 | 5.94 | 167.3 | 7.75 | *T’*(Mn^2+^)+*T’*(HCOO^-^) |
| 153.3 | 6.54 | 152.4 | 6.07 | 150.7 | 6.68 | *L*(HCOO^-^) |
| 153.4 | 1.59 | 129.4 | 6.52 | 154.5 | 2.84 | *L*(HCOO^-^)+*T’*(FMD^+^) |
| 116.5 | 6.86 | 125.2 | 3.76 | 100.3 | 6.42 | *L*(HCOO^-^)+*L*(FMD^+^) |
| 109.5 | 0.42 | 89.1 | 4.98 | 95.8 | 3.81 | *L*(HCOO^-^) |
| 93.4 | 12.97 |  |  |  |  | *L*(HCOO^-^) |
| 79.5 | 1.39 | 60.7 | 5.02 | 87.8 | 0.74 | *L*(HCOO^-^) |
| 69.2 | -0.34 | 72.8 | -0.78 | 67.7 | 0.09 | *L*(HCOO^-^) |

**ν*, *δ*, *τ*, *T’* and *L* denote stretching, bending, twisting, translational and librational modes, respectively

**Supplementary Table 12.** Compressibility related to crystallographic axes calculated for the phase II calculated in the range between 0.1 MPa and 0.86 GPa for AceMn.

| Axes | *K*(TPa^-1^) | *σK*(TPa^-1^) | Direction | | | Empirical parameters | | | |
| --- | --- | --- | --- | --- | --- | --- | --- | --- | --- |
|  |  |  | *a* | *b* | *c* | *ε*_0_ | *λ* | *P*_c_ | *ν* |
| X_1_ | 19.2724 | 2.1587 | 0.8768 | 0.0 | 0.4809 | -0.0 | -0.0264 | -0.0 | 0.4844 |
| X_2_ | 12.8293 | 1.2744 | -0.0 | 1.0 | -0.0 | -0.0 | -0.0168 | -0.0 | 0.5227 |
| X_3_ | -9.6919 | 0.3184 | -0.5031 | -0.0 | 0.8642 | 0.0 | 0.0121 | -0.0 | 0.567 |
| V | 33.8094 | 3.6679 |  |  |  |  |  |  |  |

**Supplementary Table 13.** Birch-Murnaghan Coefficients for AceMn based on data between 0.1 MPa and 0.86 GPa.

|  | *B*_0_ (GPa) | *σB*_0_ (GPa) | *V*_0_ (Å^3^) | *σV*_0_ (Å^3^) | [*B*'](http://pascal.chem.ox.ac.uk/cgi-bin/first.cgi) | [*σB*'](http://pascal.chem.ox.ac.uk/cgi-bin/first.cgi) | [*P*_c_ (GPa)](http://pascal.chem.ox.ac.uk/cgi-bin/first.cgi) |
| --- | --- | --- | --- | --- | --- | --- | --- |
| 2nd | 26.1161 | 4.7103 | 949.7571 | 3.6819 | 4.0 | n/a | 0.0 |
| 3rd | 11.5284 | 27.6856 | 950.4067 | 10.0935 | 92.7496 | 322.9742 | 0.0 |

**Supplementary Table 14.** Compressibility related to crystallographic axes calculated for the phase III calculated between 0.95 GPa and 4.1 GPa for AceMn.

| Axes | *K*(TPa^-1^) | *σK*(TPa^-1^) | Direction | | | Empirical parameters | | | |
| --- | --- | --- | --- | --- | --- | --- | --- | --- | --- |
|  |  |  | *a* | *b* | *c* | *ε*_0_ | *λ* | *P*_c_ | *ν* |
| X_1_ | 6.4698 | 0.5949 | -0.0 | -0.0 | 1.0 | 64.5014 | -64.5025 | 0.0453 | 0.0003 |
| X_2_ | 11.8842 | 1.7812 | 1.0 | 0.0 | -0.0 | 0.0673 | -0.0455 | -0.9962 | 0.5138 |
| X_3_ | 13.8829 | 1.2031 | 0.0 | 1.0 | -0.0 | 77.8426 | -77.7938 | -1.163 | 0.0007 |
| V | 26.9607 | 2.2944 |  |  |  |  |  |  |  |

**Supplementary Table 15.** Birch-Murnaghan Coefficients for AceMn calculated based on data between 0.95 GPa and 4.1 GPa.

|  | *B*_0_ (GPa) | *σB*_0_ (GPa) | *V*_0_ (Å^3^) | *σV*_0_ (Å^3^) | [*B*'](http://pascal.chem.ox.ac.uk/cgi-bin/first.cgi) | [*σB*'](http://pascal.chem.ox.ac.uk/cgi-bin/first.cgi) | [*P*_c_ (GPa)](http://pascal.chem.ox.ac.uk/cgi-bin/first.cgi) |
| --- | --- | --- | --- | --- | --- | --- | --- |
| 2nd | 19.9989 | 1.488 | 1868.0692 | 16.6566 | 4.0 | n/a | 0.0 |
| 3rd | 0.0436 | 994.8926 | 2117.0755 | 39058.9417 | 1229.7907 | 27817707.5501 | 0.0 |

**Supplementary References**

1. Havriliak, S. & Negami, S. A complex plane representation of dielectric and mechanical relaxation processes in some polymers. *Polymer (Guildf).* **8**, 161–210 (1967).

2. Wübbenhorst, M. & van Turnhout, J. Analysis of complex dielectric spectra. I. One-dimensional derivative techniques and three-dimensional modelling. *J. Non. Cryst. Solids* **305**, 40–49 (2002).

3. Mączka, M. *et al.* Perovskite Metal Formate Framework of [NH_2_-CH^+^-NH_2_]Mn(HCOO)_3_]: Phase Transition, Magnetic, Dielectric, and Phonon Properties. *Inorg. Chem.* **53**, 5260–5268 (2014).

4. Mączka, M. *et al.* Synthesis and temperature-dependent studies of a perovskite-like manganese formate framework templated with protonated acetamidine. *Dalt. Trans.* **46**, 8476–8485 (2017).

5. Nowok, A. *et al.* From ambient- to high-pressure dielectric response of perovskite formamidinium manganese formate. *J. Mater. Chem. C* **9**, 5740–5748 (2021).

6. Nowok, A. *et al.* Toward the Undiscovered Dielectric Properties of Hybrid Acetamidinium Manganese Formate under High Pressure. *J. Phys. Chem. C* **125**, 908–914 (2021).
